# Regulation of nerve cells using conductive nanofibrous scaffolds for controlled release of *Lycium barbarum* polysaccharides and nerve growth factor

**DOI:** 10.1093/rb/rbad038

**Published:** 2023-04-20

**Authors:** Jing Wang, Yuan Liu, Minmin Lv, Xiaoli Zhao, Kwok Fai So, Hui Li, Mohamed EL-Newehy, Hany EL-Hamshary, Yosry Morsi, Xiumei Mo

**Affiliations:** Research Center for Human Tissues and Organs Degeneration, Institute of Biomedicine and Biotechnology, Shenzhen Institutes of Advanced Technology, Chinese Academy of Sciences, Shenzhen, 518055, P.R. China; Department of Orthopedics, Shanghai Sixth People's Hospital, Shanghai, 201306, P.R. China; Research Center for Human Tissues and Organs Degeneration, Institute of Biomedicine and Biotechnology, Shenzhen Institutes of Advanced Technology, Chinese Academy of Sciences, Shenzhen, 518055, P.R. China; University of Hong Kong-Shenzhen Hospital, Shenzhen, 518053, P.R. China; Research Center for Human Tissues and Organs Degeneration, Institute of Biomedicine and Biotechnology, Shenzhen Institutes of Advanced Technology, Chinese Academy of Sciences, Shenzhen, 518055, P.R. China; State Key Laboratory of Brain and Cognitive Sciences, The University of Hong Kong, Pokfulam, Hong Kong, P.R. China; Department of Ophthalmology, The University of Hong Kong, Pokfulam, Hong Kong, P.R. China; Guangdong-Hong Kong-Macau Institute of CNS Regeneration, Jinan University, Guangzhou, P.R. China; Research Center for Human Tissues and Organs Degeneration, Institute of Biomedicine and Biotechnology, Shenzhen Institutes of Advanced Technology, Chinese Academy of Sciences, Shenzhen, 518055, P.R. China; Department of Chemistry, College of Science, King Saud University, Riyadh 11451, P.O. Box 2455, Saudi Arabia; Department of Chemistry, College of Science, King Saud University, Riyadh 11451, P.O. Box 2455, Saudi Arabia; Faculty of Engineering and Industrial Sciences, Swinburne University of Technology, Boroondara, VIC 3122, Australia; State Key Laboratory for Modification of Chemical Fibers and Polymer Materials, Shanghai Engineering Research Center of Nano-Biomaterials and Regenerative Medicine, College of Biological Science and Medical Engineering, Donghua University, Songjiang, Shanghai, 201600, P.R. China

**Keywords:** conductive nanofibrous scaffold, LBP, NGF, nerve cells, electrical stimulation

## Abstract

Currently, more and more patients suffer from peripheral nerve injury due to trauma, tumor and other causes worldwide. Biomaterial-based nerve conduits are increasingly recognized as a potential alternative to nerve autografts for the treatment of peripheral nerve injury. However, an ideal nerve conduit must offer topological guidance and biochemical and electrical signal transduction mechanisms. In this work, aligned conductive nanofibrous scaffolds comprising polylactic-co-glycolic acid and multiwalled carbon nanotubes (MWCNTs) were fabricated via coaxial electrospinning, and nerve growth factor (NGF) and Lycium barbarum polysaccharides (LBP) purified from the wolfberry were loaded on the core and shell layers of the nanofibers, respectively. LBP were confirmed to accelerate long-distance axon regeneration after severe peripheral nerve injury. In addition, the synergistic promotion of LBP and NGF on nerve cell proliferation and neurite outgrowth was demonstrated. MWCNTs were introduced into the aligned fibers to further increase the electrical conductivity, which promoted the directional growth and neurite extension of neurons *in vitro*. Further, the combination of conductive fibrous scaffolds with electrical stimulation that mimics endogenous electric fields significantly promoted the differentiation of PC12 cells and the axon outgrowth of neurons. Based on robust cell-induced behaviors, conductive composite fibers with optimized fiber alignment may be used for the promotion of nerve recovery.

## Graphical abstract

**Figure:**
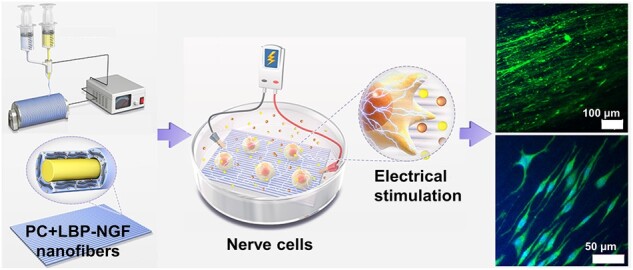

## Introduction

Peripheral nerve injury (PNI) caused by trauma, diseases or genetics may lead to partial or total loss of motor function and sensory perception because of the limited repair and functional recovery of mammalian nerve cells [1]. Approximately 2–5% of trauma patients are affected by PNI annually [2]. Current clinical treatments for PNI include surgical end-to-end coaptation, autologous nerve grafts, allografts and nerve conduits [3]. Among these procedures, autologous nerve transplantation is accepted as the ‘gold standard’ for the treatment of long-distance nerve defects [4]. However, this technique is limited by the shortage of sources, the morbidity of the donor site, the dimensional mismatch between the graft and host nerves and other potential complications. The repair of long-distance nerve defects is still one of the great challenges in PNI.

Engineered nerve guidance channels (NGCs) have been developed to guide nerve regeneration and bridge nerve defects to overcome the limitations of autologous nerve transplantation [5]. However, they did not provide sufficient regeneration for long-distance nerve defects *in vivo*. Neural tissue regeneration takes a relatively long time depending on the extent of the defect, and there might be considerable atrophy of the denervated tissue before the regenerated axons reach the distal nerve tissue [6]. Therefore, accelerating nerve regeneration is important for the recovery of peripheral nerves. By mimicking the natural extracellular matrix by integrating multiple physical cues and biomolecular signals, NGCs can create a suitable environment to promote nerve regeneration [6, 7].

Extracellular topography has been demonstrated to affect cell behavior and fate [8, 9]. Axonal bundles with highly aligned structures play a key role in the directional transmission of nerve impulses [10]. Therefore, artificial NGCs with aligned nanofibers on the inner surface can guide Schwann cell alignment and axon remyelination and promote nerve regeneration [11–13]. Electrospinning has been widely used to fabricate NGCs with aligned nano/micro-fibrous structures to direct neuronal outgrowth and extension [14, 15].

Neurotrophic factors and neurotrophins play an essential role in nerve regeneration and have been widely used in neural tissue engineering. Nerve growth factor (NGF) is the most studied neurotrophic factor as it is essential for the survival of neurons and the extension of axons [16]. Some small molecules have also been shown to orchestrate multiple signaling pathways to ‘switch on’ the intrinsic growth program of injured neurons to treat neurological damage. Lycium barbarum polysaccharides (LBP), purified from the wolfberry, have been used as a neuroprotective agent after nerve injuries [17, 18]. Studies have proved that LBP accelerate long-distance axonal regeneration after severe PNI and optic nerve crush (ONC). LBP not only promote the intrinsic growth capacity of injured neurons and functional recovery after severe PNI but also induce robust retinal ganglion cell survival and axonal regeneration after ONC [18]. Our previous studies also confirmed that LBP have positive effects on neural cell proliferation and differentiation *in vitro* and that LBP and NGF have beneficial synergistic effects in promoting neural cell differentiation and neuronal axon extension [17]. The incorporation of NGF in nerve conduits increases the number of mature nerve fibers and the diameter of the axons and enhances function recovery, resulting in increased peripheral nerve regeneration across a gap [19, 20]. However, the application of NGF is limited by its short half-life and unstable properties. The physical adsorption of NGF in conduits does not provide sustained release. Moreover, the direct incorporation of NGF in the polymer matrix during electrospinning can cause protein denaturation and reduced biological effects [21]. Besides, combined therapy with drugs and growth factors requires the optimization of their release profiles to promote tissue regeneration. Coaxial electrospinning can realize the simultaneous loading of various drugs in different layers in one step and protect delicate biomolecules from harsh environments [22, 23]. By tuning the thickness of the sheath layer and the diameter of the fiber, the release of loaded drugs can be finely tailored in a controlled and prolonged manner. Moreover, the core–shell structure of nanofibers fabricated by coaxial electrospinning can realize the loading of more than two bioactive factors or drugs and regulate their release.

Alongside topography and biochemical cues, electrical stimulation (ES) also presents dramatic effects on nerve regeneration [24, 25]. The nervous system has the strongest electrical activity, which transports signals from neurons to the desired regions through synapses. The appropriate intensity of ES can promote neurite extension and neural differentiation [26]. Conductive materials can enhance intercellular charge transportation and electrical signal transmission, which can enhance neural cell adhesion, proliferation, infiltration and migration, as well as neurite outgrowth [27]. Therefore, electrically conductive materials have been used as fillers in composite nerve conduits to promote nerve regeneration, such as polypyrrole, polythiophene, polyaniline and poly(3,4-ethylenedioxythiophene), as well as carbon nanomaterials, such as carbon nanotubes (CNTs) and graphene oxide. Biodegradable NGCs containing carbon nanomaterials became more popular in peripheral nerve regeneration compared to conductive polymers, which require complex synthesis and doping steps [28]. CNTs are advantageous owing to their high Young’s modulus and strength, good flexibility and excellent electrical conductivity. More importantly, CNTs can be functionalized by incorporating various surface groups and grafting to improve their dispersion and reduce cytotoxicity [29]. CNTs play an important role in mediating interactions between neurons and their environment. When used as scaffolds, CNTs not only act as a reservoir for adsorbed proteins but also play a dynamic role in improving the electrical properties of neurons. We explored the effects of carboxyl-modified multiwalled carbon nanotubes (MWCNTs) and exogenous ES on nerve cells in our previous study and confirmed that they promote nerve regeneration [30].

An ideal nerve conduit not only mimics the natural neural architecture but also provides a highly permissive and proregenerative microenvironment for the reconstruction of injured nerves owing to its microarchitecture. Combination therapies that involve different aspects of injury- and regeneration-associated microenvironments are key to improving the efficacy of nerve regeneration [31]. Therefore, to endow nerve repair scaffolds with enhanced proregeneration functionality and overcome their structural and functional deficiencies, we integrated multiple physical cues, biomolecular signals, as well as electrical signals to improve nerve repair and functional recovery. We prepared conductive nanofibrous scaffolds containing MWCNTs using coaxial electrospinning and loaded NGF and LBP on the core and shell layers of the nanofibers, respectively. This nanofibrous scaffold integrated multiple functions, not only as a scaffold to provide effective support and contact guidance for cells but also to synergistically release LBP and NGF at the injured site, inducing the growth of nerve synapses, to further enhance nerve regeneration.

## Materials and methods

### Materials

Polylactic-co-glycolic acid (PLGA) (lactide/glycolide: 75/25 in molar ratio; inherent viscosity: 0.61 dl/g) was purchased from Jinan Daigang Biomaterial Co., Ltd, China. Carboxyl-modified MWCNTs (outer diameter: 10–20 nm; length: 1–30 μm; purity: >99.9 wt%; COOH content: 1.0–2.0 wt%) were purchased from Cheap Tubes Inc., Cambridgeport, VT, USA. LBP were provided by Professor Kwok Fai So of the Guangdong-Hongkong-Macau Institute of CNS Regeneration, Jinan University. 1,1,1,3,3,3-hexafluoro-2-propanol (HFIP), glutaraldehyde and Dulbecco’s modified Eagle’s medium (DMEM/F12) were obtained from Sigma-Aldrich. Rat PC12 cells in the adherent type (ATCC^®^ CRL-1721.1) and rat Schwann cells (S42 ATCC^®^ CRL2942™) were obtained from ATCC, USA. Fetal bovine serum (FBS), horse serum (HS), trypsin/EDTA, NGF, neurobasal medium and B27 supplement factors were purchased from GIBCO Invitrogen, USA. The Schwann cell medium was purchased from Gene-Ethics Asia Pte Ltd., Singapore.

### Fabrication of aligned PLGA/MWCNTs composite nanofibers

PLGA (1.25 g) and LBP (10 mg) were simultaneously dissolved in 5 ml of HFIP. The mixed solution was placed on the magnetic stirrer and stirred for 24 h to form a uniform solution. Then, 0.1 g of carboxyl-modified MWCNTs (accounting for 8% of PLGA by mass) and 100 μL of Span 80 were added to the solution, followed by magnetic stirring for 8 h for the uniform dispersion of MWCNTs in the solution. The solution was ultrasonicated for 1 h before electrospinning. Ten  μg/ml of NGF aqueous solution was prepared, and bovine serum albumin (BSA) (with a concentration of 10 mg/ml) was added as a stabilizer to protect the activity of NGF. The aligned PLGA/MWCNTs nanofibers with shell-loaded LBP and core-loaded NGF were fabricated via coaxial electrospinning. A voltage potential of 18 kV was applied during the electrospinning process. The flow rate of the shell and core solution was set to 1 ml/h and 0.2 ml/h, respectively, and the distance between the needle and the collector was set to 10 cm. A high-speed rotating (4000 rpm) roller wrapped in aluminum foil was used to collect aligned nanofibers (PC + LBP-NGF). The aligned PLGA/MWCNTs nanofibers without LBP and NGF and with only NGF (PC and PC-NGF, respectively) were also fabricated as the controls.

### Characterization of PLGA/MWCNTs composite nanofibers

The surface morphologies of the electrospun PC, PC-NGF and PC + LBP-NGF nanofibers were evaluated by scanning electron microscopy (SEM). The hydrophilicity of the electrospun nanofibers was measured by water contact angle measurements using a VCA Optima surface analysis system (AST products, Billerica, MA, USA). The tensile properties of the nanofibrous scaffolds were evaluated using a universal materials tester (H5K-S, Hounsfield, UK) at an ambient temperature of 20°C and humidity of 65%. Prior to tensile testing, the thickness of the specimens was measured with a digital micrometer (Digimatic Micrometer Series 293 MDC-MX Lite, Mitutoyo, Kawasaki, Japan). Mechanical testing was conducted on specimens with a thickness in the range of 60–80 μm with a stretch speed of 10 mm/min.

### Conductivity of PLGA/MWCNTs composite nanofibers

To evaluate the effects of LBP and NGF on the conductivity of PLGA/MWCNTs nanofibers, the conductivities of PC, PC-NGF and PC + LBP-NGF nanofibers were measured along the fiber orientation. The fiber mat was cut into rectangles (10 mm × 20 mm) with the fiber orientation direction along the long axis of the rectangle. The thickness of the fiber film was measured with a spiral micrometer. The resistance of the fiber mat was measured with an ohmmeter, and the conductivity was calculated with the following formula: where *R* is the measured resistance, *L* is the length of the test sample and *S* is the cross-sectional area of the test sample. *ρ* and *κ* represent the resistivity and conductivity, respectively. Six samples were measured for each fibrous scaffold, and the average value was calculated.


R=(ρL)/S  and  κ=1/ρ.

### Release behavior of LBP and NGF *in vitro*

The fabricated PC + LBP-NGF nanofibrous mat was cut into small pieces, and samples with a weight of approximately 50 mg were placed into 2-ml centrifuge tubes, followed by the addition of 1 ml of PBS solution (pH = 7.4). The centrifuge tubes containing the nanofibrous mats were put into a shaker at 37°C with a shaking rate of 100 rpm to release LBP from the nanofibrous mats into the PBS solution. PBS was collected and replaced at preset time points. The collected PBS was stored at −20°C for later tests. The released amounts of LBP at each time point from the nanofibrous mats were analyzed by a UV–vis spectrophotometer at a wavelength of 260 nm, as previously described [17].

The *in vitro* release of NGF from PC + LBP-NGF and PC-NGF nanofibrous mats was determined. The fibrous mats were cut into small pieces and samples with a weight of approximately 50 mg were soaked in 1 ml PBS (pH = 7.4). The samples were further incubated in a shaker at 37°C with a shaking rate of 100 rpm. The PBS solutions containing released NGF were also collected and stored at −20°C at each preset time point. The amount of NGF released at each time point was measured using the enzyme-linked immunosorbent assay (ELISA).

### Degradation study

To perform the degradation study, the fabricated nanofibrous mats were cut into small square pieces and precisely weighed (*W*_0_), followed by immersion in 10 ml of PBS at 37°C under continuous shaking (150 rpm) for 7 weeks. PBS was changed once a week to avoid pH fluctuations due to degradation products. At predetermined intervals, the specimens were taken out from PBS, washed with deionized water, dried in a vacuum and weighed (*Wt*). The weight loss (%) of each sample was further calculated using the formula (*W*_0_ −*Wt*)/*W*_0_ × 100%. Furthermore, the morphology of each sample was observed using SEM.

### *In vitro* cell culture

S42 cells (rat Schwann cells) were cultured in DMEM (high glucose) supplemented with 10% FBS and 1% penicillin/streptomycin solution. PC12 cells were cultured in the normal growth medium composed of DMEM/F12 supplemented with 10% HS, 5% FBS and 1% antibiotic/antimycotic solution. All cells were maintained in a humid incubator at 37°C with 5% CO_2_ supply. The nanofibrous mats for cell culture were collected using 15-mm coverslips and placed into 24-well cell culture plates. The samples in each well were soaked in 75% alcohol, and the plates were exposed to ultraviolet light for 5 h. After sterilization, the samples were washed three times with PBS, followed by immersion overnight in the growth media of S42 and PC12 cells.

### *In vitro* cell proliferation

S42 and PC12 cells were seeded on each sample at a density of 10^4^ cells per well. The proliferation behaviors of S42 and PC12 cells on various fibrous mats were monitored using the Alamar Blue assay according to the instructions of the manufacturer. After 1, 4 and 7 days of cell seeding, 10% Alamar Blue was added to each well, followed by incubation in 5% CO_2_ at 37°C for 4 h. Then, the fluorescence intensity was measured using a Varioskan flash reader (Thermo Fisher Scientific, MA, USA) at 560 nm (excitation)/590 nm (emission).

### S42 cell morphology

After 7 days of culture, S42 cells cultured in each well were rinsed twice with PBS and then fixed with formalin for 20 min at room temperature (25°C). After that, the cell/nanofiber complexes were rinsed three times with distilled water, further dehydrated using a series of graded ethanol (50, 70, 90 and 100%, for 15 min for each concentration of ethanol), and naturally dried. After the samples were sputter-coated with gold, they were observed under SEM.

Furthermore, to illustrate the effects of various fibrous mats on S42 cell maturity, immunofluorescent staining with S100 was conducted. After 7 days of culture, cells on different fibrous mats were fixed with formalin for 20 min, permeabilized in 0.1% Triton X-100 solution for 10 min, and blocked in 3% w/v BSA aqueous solution for 90 min. The cells were treated with an anti-S100 antibody produced in rabbits (diluted at 1:100, Sigma) for 2 h at room temperature. Secondary antibody labeling was performed with FITC-conjugated goat anti-rabbit (Sigma) at a dilution of 1:300 for 1 h. After further treatment with mounting medium with DAPI (Vector Laboratories, USA), the samples were characterized using confocal laser scanning microscopy (CLSM, Zeiss LSM700).

### *In vitro* differentiation of PC12 cells

To examine the bioactivity of NGF released from the scaffolds and the synergistic induction effects of LBP and NGF on PC12 cell differentiation, the differentiation behavior of PC12 cells in different scaffolds and under different differentiation conditions was characterized. PC12 cells were seeded on different nanofibrous mats placed at the bottom of 24-well plates at a density of 2 × 10^4^ cells per well according to the cell seeding method mentioned above. After 24 h of culture, to allow cells to fully adhere to the scaffolds, the cell culture medium was changed to a different differentiation medium. The differentiation medium DMEM/F12 supplemented with 1% HS, 0.5% FBS and 1% antibiotic/antimitotic solution containing 100 ng/ml NGF (NGF^+^) was used for cells cultured on PC nanofibrous mats, whereas the differentiation medium without NGF (NGF^−^) was used for cells cultured on PC-NGF and PC + LBP-NGF nanofibrous mats. After 7 days of culture, the cells were fixed with formalin and dehydrated with graded ethanol following the method used above, and the morphology after differentiation induction on different fibrous mats was observed with SEM.

Additionally, the morphologies of PC12 cells were detected by immunofluorescent staining after 7 days of differentiation induction. Tubulins are cytoskeletal proteins, so the microtopography of cells can be observed indirectly by observing tubulin distribution; then, the cellular topography of cells after differentiation on different scaffolds can be comparatively studied. The detailed procedure for the immunostaining of α-tubulin was the same as described above. The cells were sequentially treated with mouse anti-α-tubulin (Sigma), secondary antibodies with the goat anti-mouse antibodies (IgG H + L, Alexa Fluor 488, Abcam) and DAPI. Finally, the samples were visualized under CLSM to observe the neurite extension of differentiated PC12 cells.

### Differentiation of PC12 cells on a conductive fibrous mat under ES

The differentiation behavior of PC12 cells on conductive fibrous mats loaded with LBP and NGF under external ES was further investigated. PC12 cells were seeded on PC + LBP-NGF nanofibrous mats in a 24-well plate at a density of 2 × 10^4^ cells per well. After 24 h of culture, the medium was replaced with the differentiation medium without NGF. Meanwhile, cells on the fibrous mat were electrically stimulated daily for 1 h, and the fresh differentiation medium without NGF was used after ES. In detail, two electrodes were inserted into the opposite ends of the fibrous mat seeded with cells to expose them to trains of electrical pulses (rectangular, 40 mV) for 30 min [25]. The direction of the electric pulse was consistent with the axial direction of the aligned nanofibers. After 7 days of differentiation induction under ES, the differentiation of PC12 cells was identified using SEM and immunofluorescence staining with α-tubulin. The neurite length of differentiated cells was counted using Image J software from the corresponding fluorescence micrograph. The neurite length of PC12 cells was defined as the distance from the tip of a neurite to the junction between the neurite base and the cell body, and the value was expressed relative to the respective cell soma [32].

### Axon extension of dorsal root ganglia neurons on a conductive fibrous mat under ES

To examine the effects of the external ES on neurite outgrowth, dorsal root ganglia (DRG) neurons were used. DRG neurons were isolated from the embryos of 14 days pregnant rats according to the methods used in our previous research [30]. First, embryos were placed on a 10-cm Petri dish, and spinal cords were collected. Spinal cords were then placed in clean Petri dishes. After that, spinal cord tops were fixed, and central meninges were cut from the top to the bottom; then, spinal cords were turned over, and meninges were stripped off with DRG neurons. Subsequently, the tissue was digested in 0.25% trypsin and mechanically dissociated in the neurobasal medium using a pipette. The harvested neurons were seeded on fibrous mats placed in a 24-well plate at a density of 10^5^ neurons per well. After 24 h of culture in the neurobasal medium supplied with 1% penicillin–streptomycin, 2% B27 and 20 ng/ml NGF, no more NGF was added to the medium. During the following 7 days of culture, ES was applied for 30 min to the DRG neurons on the fibrous mats per day using the same electrical pulses mentioned above. Thereafter, the neurons were fixed with 4% paraformaldehyde for 30 min at 4°C, rinsed with PBS, permeabilized with 0.1 wt% Triton X-100 for 10 min and blocked with 10 wt% BSA in PBS for 30 min. The neurons were immunostained with anti-160 kDa neurofilament (NF160) primary antibody (Abcam) and goat anti-rabbit IgG Alexa 488-second antibody (Invitrogen). Fluorescence micrographs were captured using CLSM.

### Statistical analysis

All the quantification data were presented as mean ± standard deviation. Statistics for multiple comparisons were generated using one-way analysis of variance followed by Student’s *t*-test. *P <* *0.05, **P <* *0.01 and ***P <* *0.001 were considered statistically significant.

## Results and discussion

### Characterization of fabricated nanofibers

**Figure 1.**
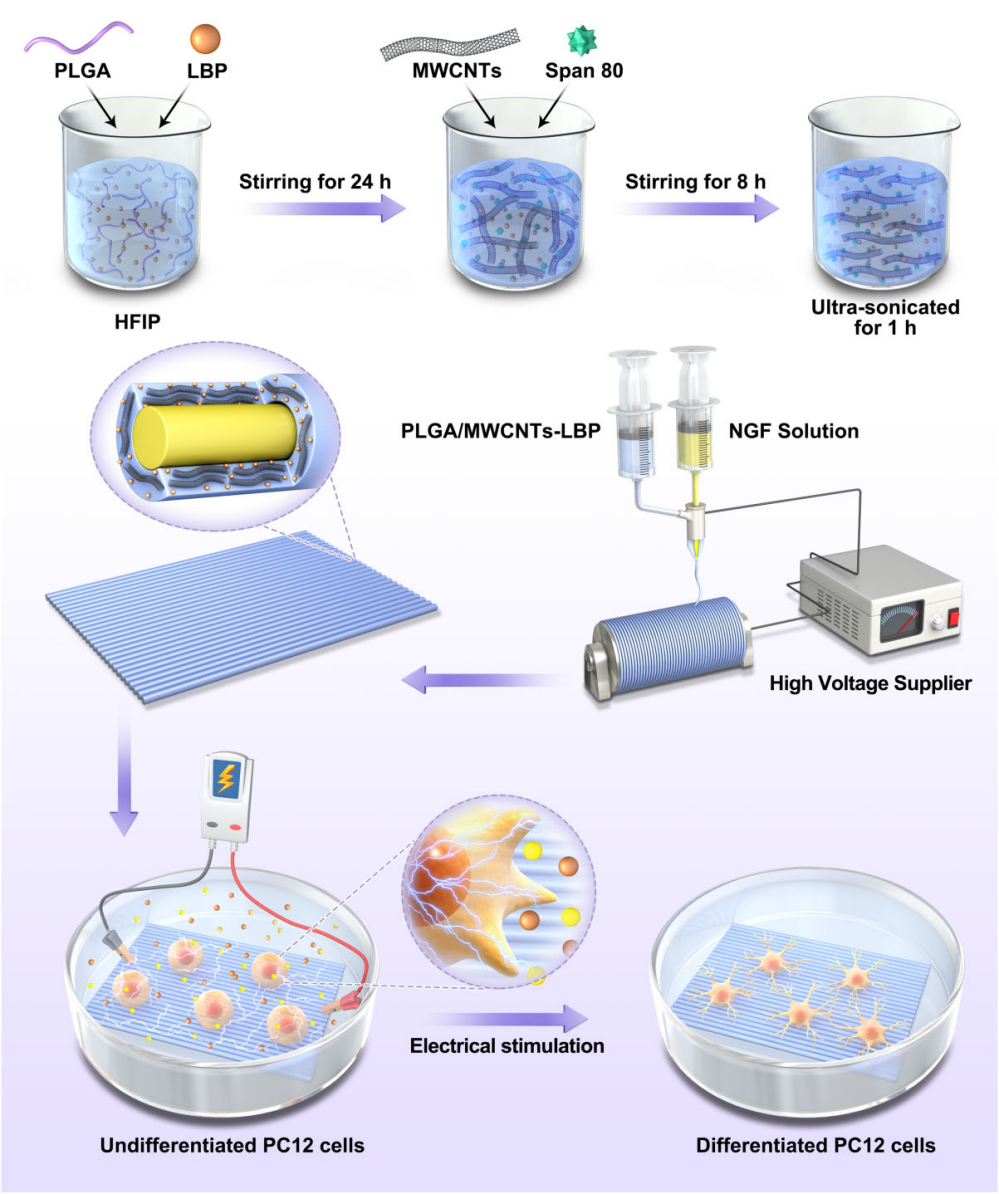
Schematic diagram of the fabrication process of conductive nanofiber scaffolds simultaneously loaded with LBP and NGF, and the regulation of nerve cells via electrical stimulation.

In this study, conductive nanofibrous scaffolds loaded with LBP and NGF were fabricated, and the fibrous scaffolds simultaneously possessed topographic guidance for cells, sustained release of LBP and NGF and electrical signals. The fabrication process of the nanofibrous scaffolds is illustrated in Fig. 1.

**Figure 2.**
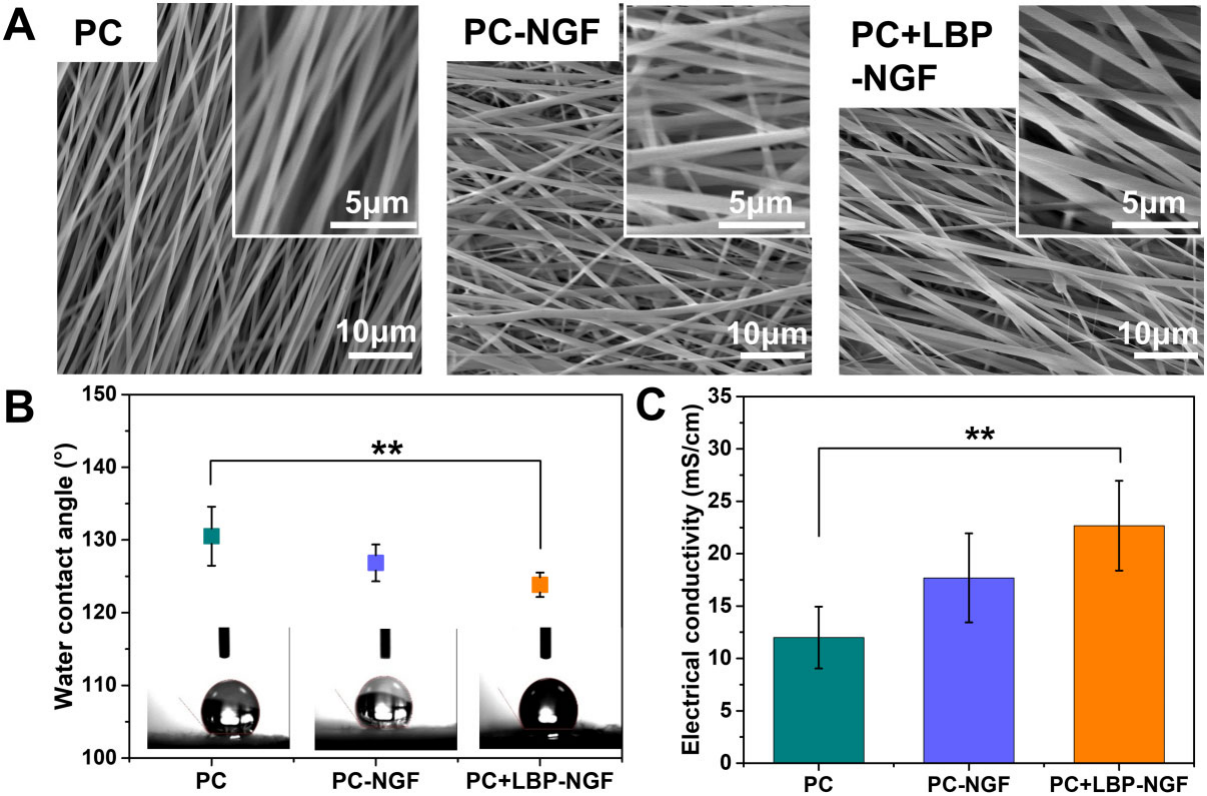
Morphology, hydrophilicity, electrical conductivity, and mechanical properties of various conductive electrospun nanofibers. (**A**) SEM morphologies, (**B**) water contact angles and (**C**) conductivities of electrospun nanofibrous mats in the direction parallel to the fiber orientation. **P <* *0.01.

Our previous work showed that the addition of 8 wt% MWCNTs (accounting for the mass ratio of the polymer) endowed the polymer fibers with enhanced electrical conductivity and the ability to promote cell proliferation [33]. Thus, conductive composite fibers were prepared using 8 wt% MWCNTs in this study. In addition, carboxyl-functionalized MWCNTs were used owing to their increased hydrophilicity and minimized toxicity compared to pristine MWCNTs. The morphologies of the fabricated nanofibers were evaluated using SEM. As shown in Fig. 2A, these fibers exhibited a smooth surface and bead-free morphology, showing parallel alignment. MWCNTs were evenly distributed in the fibers, and no obvious agglomeration was observed. Their average fiber diameters were comparable and not significantly influenced by the incorporation of LBP and NGF.

The water contact angles of three different nanofibrous mats were determined to confirm the effects of LBP and NGF on their hydrophilicity, and the results are shown in Fig. 2B. The PC scaffold was demonstrated to be the most hydrophobic sample with a contact angle of 130.52 ± 4.05°. After the fiber core layer was loaded with NGF, the water contact angle on the surface of the scaffolds changed to 126.84 ± 2.53°, which was not significantly different from that of the PC scaffold. However, after increasing the loading of LBP on the fibrous shell layer, the water contact angle of the scaffolds decreased to 123.86 ± 1.67°. LBPs are water-soluble natural polysaccharides. During the preparation of the scaffolds, LBP and PLGA blends were extruded from the outer layer of coaxial needles; thus, after fiber shaping, LBPs were located in the shell layer of the fibers, and they were partially located on the surface of the nanofibers, decreasing the water contact angle of the fibers and increasing their hydrophilicity.

Notably, the electrical conductivity of scaffolds is crucial for accelerating nerve regeneration under ES. It has been reported that the aligned composite fibers containing CNTs showed anisotropic electrical conductivity, where the value in the parallel direction to the fiber axis was 10 times higher than that in the perpendicular direction [34]. This can be attributed to the fact that electrons move easily along the fiber, while their transfer in the vertical direction to the fiber axis is hindered [35]. Thus, the conductivities of the three different fibrous mats in parallel directions to the bundle axes were evaluated. As shown in Fig. 2C, the conductivities of PC, PC-NGF and PC + LBP-NGF were 11.99 ± 2.95 mS/cm, 17.68 ± 4.25 mS/cm and 22.67 ± 4.28 mS/cm, respectively. The electrical conductivity of the scaffolds along the direction of fiber orientation increased after the loading of LBP and NGF. The scaffolds displayed relatively good conductivity comparable to conductive materials. It was reported that a single-layered graphene/PCL conduit displayed a conductivity of 8.92 mS/cm [36]. Thus, this favorable electrical conductivity of our fibrous scaffold with MWCNTs laid a solid theoretical foundation for further accelerating nerve regeneration under ES.

**Figure 3.**
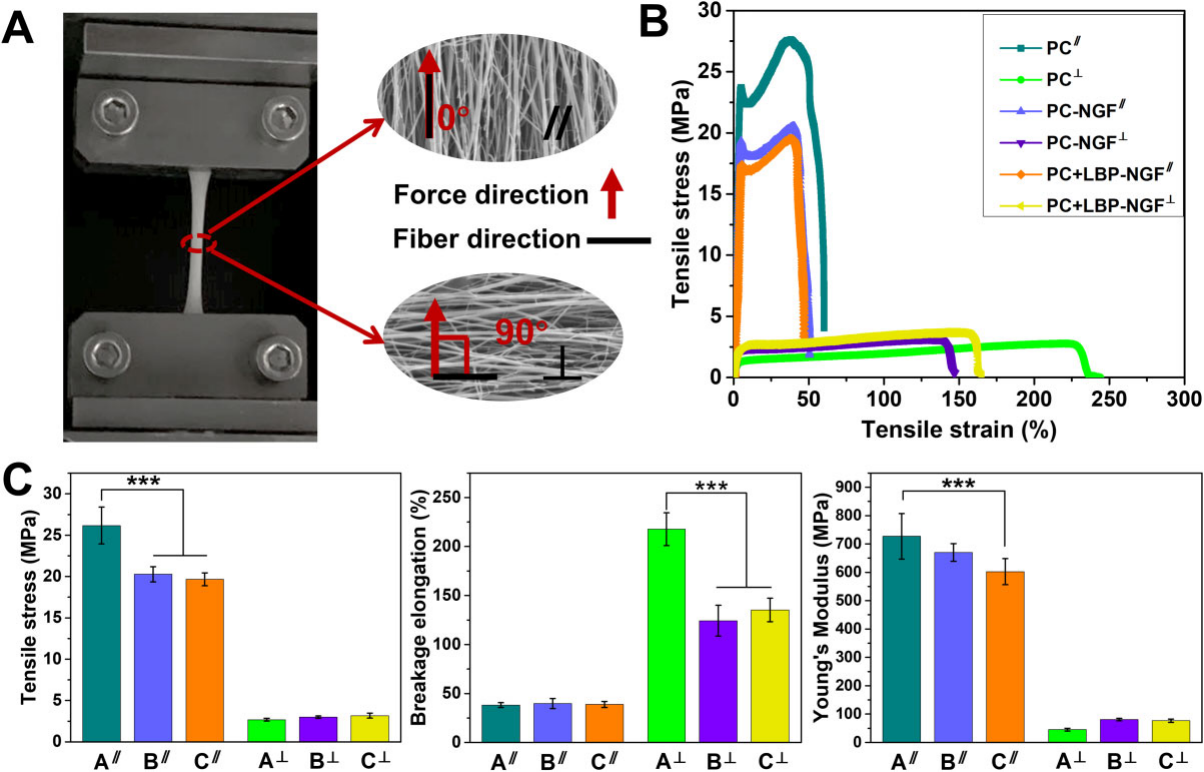
(**A**) Schematic diagram of mechanical tests performed in two directions. (**B**) Stress–strain profiles in parallel and perpendicular directions to the fiber orientation. (**C**) Tensile stress, breakage elongation and Young’s modulus in parallel and perpendicular directions to the fiber orientation. ***P <* *0.001. A: PC; B: PC-NGF; C: PC + LBP-NGF. ∥: parallel direction to the fiber orientation; ⊥: perpendicular direction to the fiber orientation.

Further, the mechanical performances of the electrospun composite fibrous mats were studied. Mechanical tests were performed parallel and perpendicular to the fiber direction, as shown in Fig. 3A. The typical stress–strain profiles for the three types of nanofibrous mats in the parallel and perpendicular directions are shown in Fig. 3B. The tensile stress, breakage elongation and Young’s modulus of the three different fibrous mats were further calculated and compared (Fig. 3C and Table 1). In comparison to the pure PC nanofibrous mat, the tensile stress of the PC-NGF fibrous mat in the direction parallel to the fiber orientation significantly decreased. After the fiber core and shell layers were loaded with NGF and LBP, respectively, both the tensile stress and Young’s modulus of the nanofibers showed a significant decrease in the direction parallel to the fiber orientation compared with those of the pure PC scaffolds, whereas the breakage elongation did not change significantly.

**Table 1.** Tensile stress, breakage elongation, and Young’s modulus of various fibrous mats

Fibrous mat	Tensile stress (MPa)	Breakage elongation (%)	Young’s modulus (MPa)
PC^a^	26.17 ± 2.24	38.16 ± 2.46	727.00 ± 80.00
PC-NGF^a^	20.27 ± 0.92	39.77 ± 5.00	670.03 ± 31.04
PC + LBP-NGF^a^	19.66 ± 0.79	38.86 ± 3.11	602.29 ± 46.15
PC^b^	2.68 ± 0.17	217.60 ± 16.77	45.40 ± 4.94
PC-NGF^b^	2.99 ± 0.14	124.24 ± 15.74	80.65 ± 4.45
PC + LBP-NGF^b^	3.17 ± 0.30	135.27 ± 11.99	76.76 ± 5.88

^a ^Parallel direction to the fiber orientation.

^b ^Perpendicular direction to the fiber orientation.

Different mechanical properties were obtained in the two examined directions for the aligned fibers. All fibrous mats exhibited lower tensile stress and Young’s modulus in the direction perpendicular to the fiber direction compared to those obtained in the parallel direction to the fiber orientation. In other words, the fibrous specimens were prone to tearing when the tensile force was applied perpendicular to the fiber axis. However, the fibrous mat exhibited a higher breakage elongation when the tensile force was applied perpendicular to the principal fiber axis. Additionally, a noticeable difference in the breakage elongation was observed between the pure PC scaffold and the PC-NGF (or PC + LBP-NGF) scaffold as the samples were loaded with a force perpendicular to the direction of the fiber axis, suggesting that NGF loading at the core layer significantly reduced the flexibility of the fibrous mat perpendicular to the fiber direction. Overall, the mechanical properties of the scaffolds decreased after the loading of LBP and NGF because they are small molecules that cannot bear a large tensile force, and they were dispersed in the PLGA fibers, affecting the continuity of PLGA. Previous studies have also shown that the addition of small-molecule drugs to fibers can have a plasticizing effect, which can reduce the tensile properties of fibrous scaffolds [37].

### Release patterns of LBP and NGF *in vitro*

**Figure 4.**
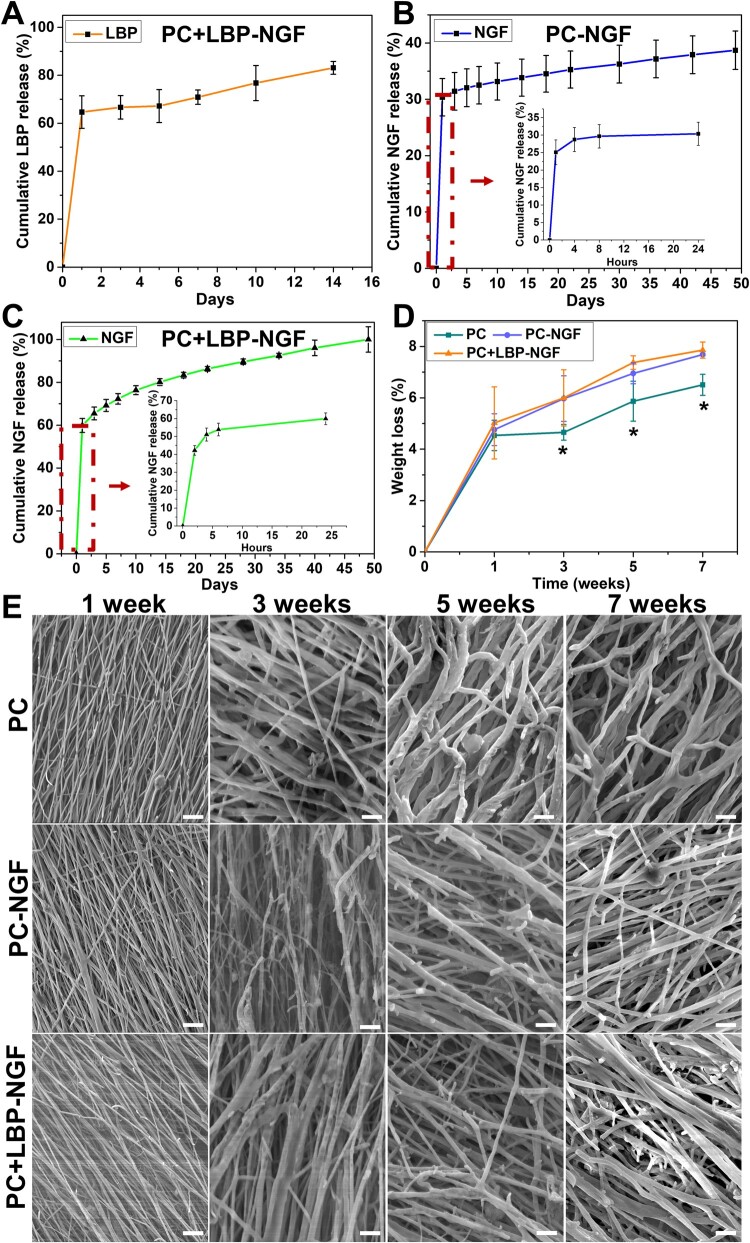
Release patterns of LBP and NGF in conducive nanofibers and the degradation of three different groups of nanofibers *in vitro*. (**A**) Cumulative release profile of LBP in PC + LBP-NGF nanofibers. (**B** and **C**) Cumulative release profile of NGF in PC-NGF and PC + LBP-NGF nanofibers, respectively. (**D**) Weight loss of three different groups of nanofibers after 7 weeks of degradation *in vitro*. (**E**) Surface morphology of three different groups of nanofibers after degradation for 1, 3, 5, and 7 weeks *in vitro* (scale bar: 10 μm).

The cumulative amount of LBP released from the PC + LBP-NGF scaffold was evaluated, and the release profile is shown in Fig. 4A. Sixty-five percent of the LBP was rapidly released during the first day, followed by a sustained, relatively slow release, and after 14 days, the cumulative release reached approximately 83%. Since LBPs were mixed in PLGA solution during the preparation of fibers, a portion of LBP was present on or near the surface of the fibers, which explains the large amount of LBP released on the first day. The amounts of NGF released from the PC-NGF and PC + LBP-NGF scaffolds were detected with the ELISA kit. After analysis and comparison, the release kinetics of NGF from the two scaffolds were found to be significantly different. Figure 4B demonstrates the release profile of NGF from the PC-NGF scaffold with a cumulative release rate of 30.37% ± 3.31% at 24 h. It was followed by a slow release process, and by Day 49, the cumulative release rate reached 38.71% ± 3.41%. However, the cumulative release percentage of NGF released from the PC + LBP-NGF scaffolds (Fig. 4C) was 59.89% ± 3.27% at 24 h and reached 100% by Day 49. NGF in the PC + LBP-NGF scaffold showed faster release compared to that in the PC-NGF scaffold, indicating that the loading of LBP in the shell layer of the fibers can accelerate the release of NGF from the core layer because holes would be left in the shell layer after the release of LBP so that water molecules would more easily enter the interior of the fiber, and NGF from the core layer would be more easily released. In addition, LBP loaded in the shell layer of the fibers could accelerate the degradation of the fibers and thus the release of NGF. Further, by comparing the release behavior of LBP and NGF in the PC + LBP-NGF scaffold, we found that LBP located in the shell layer exhibited a faster release rate than NGF located in the core layer, indicating that the release rate can be adjusted by changing the position of drug loading in the fiber (shell or core). In this study, LBP and NGF were encapsulated in the shell and core of the fibers, respectively. Therefore, the large release of LBP in the early stage can promote the rapid proliferation of glial cells, whereas the slow release of NGF can further promote the extension of synapses in the subsequent nerve repair process.

### Degradation of fibrous mats *in vitro*

After soaking in PBS at 37°C for 1, 3, 5 and 7 weeks, the weight losses of the three different fibrous mats were detected. As shown in Fig. 4D, the weight losses of the scaffolds increased gradually with the extension of the degradation time. The weight losses of the three scaffolds (PC, PC-NGF and PC + LBP-NGF) after 1 week were 4.54% ± 0.59%, 4.76% ± 0.62% and 5.02% ± 1.40%, respectively, indicating no significant differences between the three groups. After 3 weeks of degradation, the weight losses of the PC-NGF and PC + LBP-NGF scaffolds were significantly higher than that of the PC scaffold. After degradation for 7 weeks, the weight losses of PC-NGF and PC + LBP-NGF were 7.68% ± 0.04% and 7.86% ± 0.31%, respectively, whereas that of PC was 6.50% ± 0.41%, indicating that, after LBP and NGF loading, the degradation rate of the scaffolds accelerated with the release of both. This was due to the scaffolds losing part of their weight with the release of LBP and NGF. More importantly, after the release of LBP and NGF, holes would be formed in the fibers, and water molecules would more easily enter the inside of the fibers, accelerating the degradation rate of the scaffolds. On the other hand, the hydrophilicity of the PC-NGF and PC + LBP-NGF scaffolds was improved compared to the PC scaffold, which may also be one reason for the acceleration of scaffold degradation [1]. In addition, the weight loss was the greatest at week 1 for all three scaffolds, which was because the MWCNTs present on the surface of the scaffolds were water-soluble and easily dispersed from the fibrous scaffolds into PBS solution, thereby causing larger weight loss of the scaffolds.

The fiber morphologies of the three scaffolds after degradation at different times are shown in Fig. 4E. Compared with the surface morphology of the initial fibrous scaffolds, a slight fracture appeared on the fiber surface after degradation for 1 week, and the surface morphologies of the three scaffolds showed little difference. After 3 weeks of degradation, the fibers became significantly swollen and thickened. With the extension of degradation time, the fiber fracture became more obvious, and the nanofibers inside the scaffolds, especially those loaded with LBP and NGF, fractured. However, fibrous structures were retained in all three scaffolds after degradation for 7 weeks, indicating that the composite fibrous scaffold maintained its fibrous structure for 7 weeks and played a supporting role in the growth of cells.

### Cell proliferation on fibrous mats

**Figure 5.**
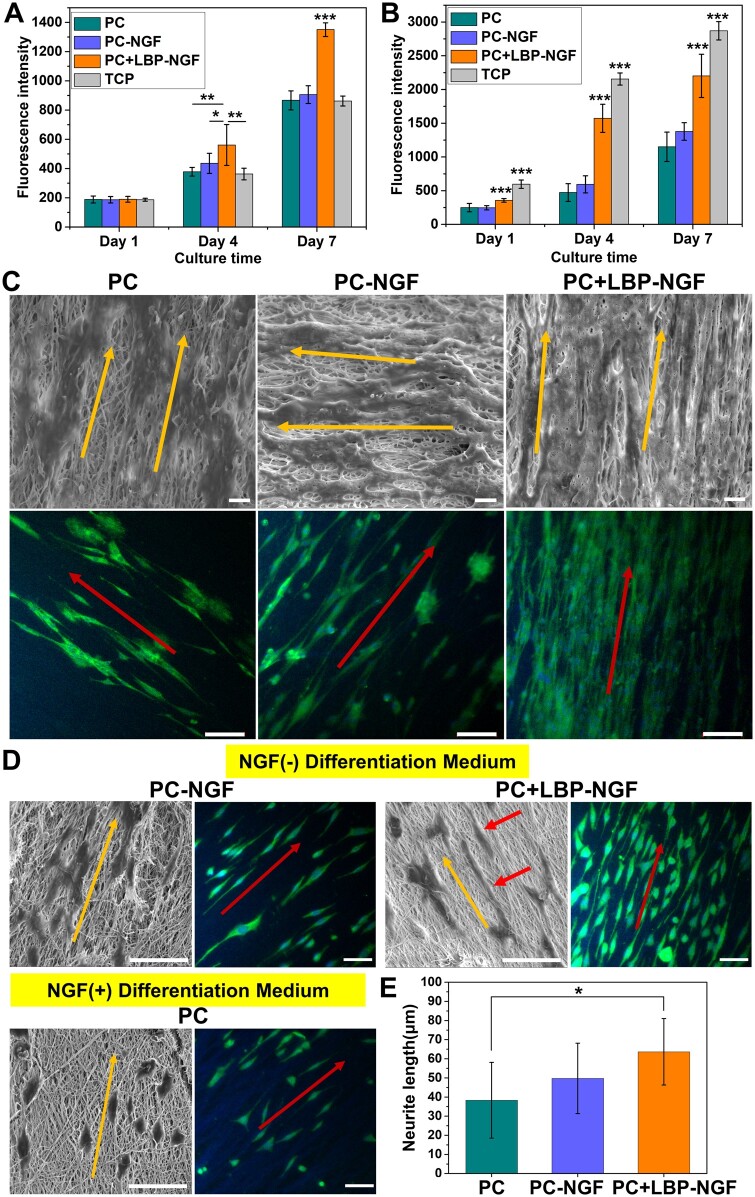
Performance of nerve cells on various electrospun conductive nanofibers. Proliferation of PC12 cells (**A**) and S42 cells (**B**) on three different groups of nanofibers. *P <* *0.05, **P <* *0.01, ***P <* *0.001. (**C**) Morphologies of S42 cells cultured on nanofibrous mats for 7 days, characterized by SEM (scale bar: 10 μm) and immunofluorescence staining (scale bar: 100 μm). The cytoplasm was stained with S100 (green), and the nuclei were stained with DAPI (blue). (**D**) Images of PC12 cells cultured on nanofibrous mats after 7 days of differentiation induction, characterized by SEM and immunofluorescence staining. Scale bar: 50 μm. (**E**) Average neurite length of differentiated PC12 cells in different fibrous scaffolds. Analysis was performed by ImageJ software, and *P* values (*P <* *0.05) are indicated on the chart.

To explore the effects of LBP and NGF loading in scaffolds on cell proliferation behavior, the proliferation of PC12 and S42 cells on the three scaffolds, PC, PC-NGF and PC + LBP-NGF, were compared. PC12 cells are widely used as neuron precursors for the study of neurogenesis. Schwann cells, as the principal glial cells in peripheral nerves, play a key role in neuron survival and regeneration [38]. As shown in Fig. 5A, the number of PC12 cells on the PC + LBP-NGF fibrous scaffolds was significantly higher than that on PC and PC-NGF scaffolds, as well as TCP after 4 days. By Day 7 of culture, the number of cells on the PC + LBP-NGF fibrous scaffolds was still the highest, and the advantage was more obvious, implying that all three scaffolds showed good biocompatibility, and LBP released from the PC + LBP-NGF scaffolds significantly promoted the proliferation of PC12 cells. Comparing the proliferation of S42 cells on three different fibrous scaffolds, we found that the results were consistent with those of PC12 cells, and the proliferation rate was the fastest on the PC + LBP-NGF scaffolds (Fig. 5B). On the contrary, PC12 cells proliferated faster on fibrous scaffolds than on TCP, whereas S42 cells proliferated faster on TCP than on fibrous scaffolds. This may be due to the hardness of the surface. It has been reported that the surface stiffness required for the growth of PC12 versus S42 cells is different, with S42 cells preferring a relatively stiff surface [39, 40]. Overall, the MWCNTs-containing scaffolds did not exhibit cytotoxicity, and the scaffolds were more favorable for the proliferation of PC12 and S42 cells after the loading of LBP. Despite some evidence of MWCNTs toxicity, many studies have demonstrated good biocompatibility of CNT materials [29, 34, 41, 42].

### S42 cell morphology on fibrous mats

The overall morphologies of S42 cells seeded on the fibrous mats after 7 days were observed using SEM, as shown in Fig. 5C. As observed, S42 cells adhered and spread well on the three scaffolds, and the direction of cell elongation was precisely parallel to the orientation direction of fibers. The number of cells was higher on the PC-NGF and PC + LBP-NGF scaffolds, especially on the PC + LBP-NGF scaffold; the cells were more tightly arranged, and they covered the entire scaffold surface. Further immunofluorescence staining showed that S42 cells expressed S100 on all three scaffolds, and the cells showed directionally arranged growth, which was consistent with SEM observations. The cells also showed distinct filopodia, which played a role in intercellular junctions. Furthermore, the number of cells within the same area was greater on the scaffolds loaded with LBP and NGF, which was consistent with the proliferation results of S42 cells. The above data indicated that the substrate structure with a longitudinally aligned topography can regulate cell growth *in vitro* through contact guidance, while LBP released from the scaffolds can promote Schwann cell growth and migration. In addition, it has been reported that the maturation of S42 cells can be promoted with the increased electrical conductivity of scaffolds [29]. Our results also confirm this conclusion as the cells on the three conductive fibrous scaffolds containing MWCNTs appeared mature, showing stretched morphologies extending along the fiber direction.

### Differentiation of PC12 cells on fibrous mats

PC12 cells are model cells commonly used for neurogenesis studies, and their morphological changes directly reflect the degree of differentiation. To explore the effectiveness of NGF loaded in the scaffolds on PC12 cell differentiation induction and the effects of released LBP on PC12 cell differentiation, the differentiation behaviors of PC12 cells on the fibrous scaffolds were compared. It is known that PC12 cells respond upon NGF induction, manifested as proliferation arrest, phenotypic changes and the acquisition of morphological characteristics and physiological properties of sympathetic neurons. Thus, the cells on the PC fibrous mat were treated with the differentiation medium containing NGF, while cells on the PC-NGF and PC + LBP-NGF fibrous mats were cultured in the differentiation medium without NGF. The cell morphology was visualized using SEM and immunofluorescence staining after 7 days of differentiation induction. As shown in Fig. 5D, cells appeared fusiform or polygonal and extended elongated neurites from the soma, indicating that both exogenously added NGF and NGF released from the scaffolds can induce the neural differentiation of PC12 cells. On the fibers aligned in parallel, the cells assumed an oriented morphology elongated along the fiber direction. Contrasting the cells on the PC and PC-NGF scaffolds revealed that the differentiation of PC12 cells on the PC-NGF scaffolds was more pronounced with cells extending longer synapses. This shows that the slowly released NGF from the scaffold has higher inducing activity compared to the exogenously added NGF. Further comparison of the cells on the PC-NGF and PC + LBP-NGF scaffolds showed that the differentiation of PC12 cells was further improved after LBP loading in the scaffolds, and the differentiated synapses extended longer. The quantification of the average neurite length further confirmed this observation. As shown in Fig. 5E, the average neurite lengths of differentiated PC12 cells on the PC, PC-NGF and PC + LBP-NGF scaffolds were 38.32 ± 19.80, 49.76 ± 18.42 and 63.64 ± 17.38 μm, respectively. In our previous study, the average neurite length of PC12 cells after 7 days of differentiation was approximately 30–40 μm in an aligned nanofibrous scaffold [43]. In this study, the average neurite length improved to approximately 60 μm after the simultaneous loading of NGF and LBP in the scaffold. As reported previously, the mean neurite length was approximately 40 μm in a hydrogel with MWCNTs after 7 days of differentiation induction [44]. Therefore, it was confirmed that LBPs have a promoting effect on NGF-induced PC12 cell differentiation and synaptic growth. Studies have shown that LBP can promote the neural differentiation of neural stem cells and inhibit the abnormal differentiation of glial cells [45, 46].

### Neurite outgrowth on fibrous mats under ES

**Figure 6.**
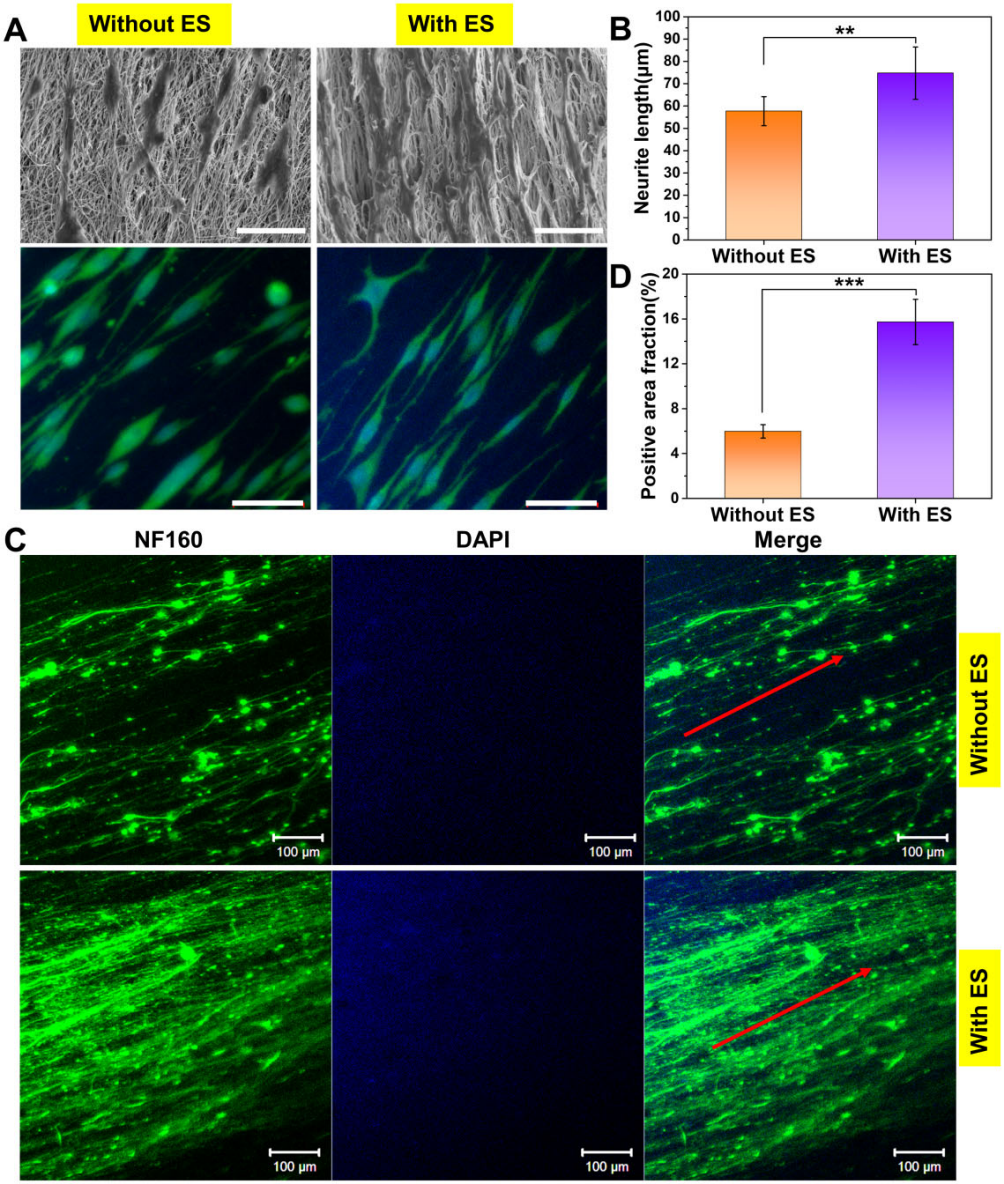
Neurite outgrowth of PC12 cells and DRG neurons on PC + LBP-NGF nanofibers under ES. (**A**) SEM images and immunofluorescent staining of PC12 cells after 7 days of differentiation under ES. The cytoskeletal protein was stained with α-tubulin (green), and the nuclei were stained with DAPI (blue) (scale bar: 50 μm). (**B**) Average neurite lengths of differentiated PC12 cells under different conditions. The analysis was performed by ImageJ software, and *P* values (**P <* *0.01) are indicated on the chart. (**C**) Immunofluorescent staining of rat DRG neurons on PC + LBP-NGF nanofibers after 7 days of culture under ES. The green color indicates the expression of NF160, and the blue color indicates the nuclei. ES: Electrical stimulation (scale bar: 100 μm). (**D**) Positive area fractions of the DRG neuron immunofluorescence images. The analysis was performed using image J software, and *P* values (***P <* *0.001) are indicated on the chart.

As electrically excitable cells, neurons are sensitive to exogenous ES and exhibit different behaviors and functions. Numerous studies have indicated that exogenous ES can accelerate axon outgrowth [47]. To investigate the application of the fabricated conductive fibrous scaffold loaded with LBP and NGF for the development of peripheral nerves, we further studied the axon outgrowth of PC12 cells and DRG neurons on the fibrous mats under ES. The differentiation and synaptic extension of PC12 cells were confirmed by cell morphology observation and immunofluorescence staining (Fig. 6A). As a result, in the NGF-free differentiation medium, PC12 cells can also differentiate toward neurons owing to the inductive effect of NGF released from the scaffold, with most cells extending long neurites, especially under the conditions of external ES, and some already differentiating multiple synapses. The average neurite length on the PC + LBP-NGF scaffolds with ES was 74.75 ± 11.69 μm, while the value without ES was about 57 μm. As reported in a recent study, the mean neurite length of PC12 cells in a conductive scaffold with ES was approximately 50–60 μm, whereas it was less than 20 μm without ES after the same differentiation culture time.[48] The results confirmed that the differentiation and neurite extension of PC12 cells improved under the combined induction of electrical signals and small molecules (LBP and NGF) released from the scaffold. This was attributed to the effects of electrical signals mediated by the conductive scaffold on the cells, as confirmed in previous studies [49, 50]. ES can trigger biophysical changes on the cell surface, affecting membrane protein functions by changing the charge distribution, such as enzyme activity, membrane receptor complexes and ion transport channels [51]. Furthermore, ES may have promoted the release of the drugs (LBP and NGF), thereby exerting an enhanced promoting effect on the cells. It has been reported that ES can promote the release of bioactive factors loaded on conductive scaffolds [52].

The axon outgrowth of DRG neurons was further detected under ES using the immunofluorescence staining of expressed NF160. As shown in Fig. 6C, NGF released from scaffolds could induce long axons to extend in the direction of scaffold fiber orientation in the NGF-free differentiation medium, and the neurites that diverged from neuronal cell bodies were denser under ES. The positive area fraction of NF160 was assessed by the immunostaining images with Image-pro plus 6.0 (Media Cybernetics, Inc., Rockville, MD, USA). As shown in Fig. 6D, the positive area fraction of DRG neurons in the scaffold with ES was much higher than that without ES, suggesting that the synergistic effects of ES and NGF induced neurons to extend more neurites and promote the extended growth of synapses. Therefore, small-molecule-loaded conductive scaffolds combined with exogenous electrical signals can promote not only neuronal differentiation but also the neurite outgrowth of primary neurons. Interactions between cells and conductive materials can occur at the material interface through physical and chemical signal recognition and transmission. Biochemical cues, such as NGF, are carried and released by scaffolds that can be detected via specific receptors on the cell membrane. Biophysical cues, such as the alignment of fibers, are inherent structures of scaffolds that can coregulate cell behavior. Furthermore, extrinsic electrical signals can manipulate the behavior of excitable cells by altering membrane biophysics. However, the mechanisms through which cells sense and respond to electrical signals remain to be further explored [53].

## Conclusions

We prepared conductive nanofiber scaffolds comprising MWCNTs for the simultaneous and controlled release of LBP and NGF. The *in vitro* release behaviors of LBP and NGF were explored to determine the sustainability of the release, and the released LBP and NGF maintained their bioactivity and function. The release of LBP promoted the proliferation of PC12 and S42 cells on the PC + LBP-NGF scaffold. In parallel, the synergistic effects of LBP and NGF enhanced PC12 cell differentiation and Schwann cell maturation. The addition of MWCNTs endowed the aligned nanofibers with conductive properties. Consequently, the fabricated composite fibrous scaffolds with optimized matrix alignment and controlled release of LBP and NGF significantly enhanced the promoted axon growth under ES. When DRG neurons were cultured for 7 days, bundled neuronal axons were observed. This scaffold possessed both topologically and electrically conductive properties in the oriented arrangement, as well as biological signals to slow release LBP and NGF, combined with external electrical signals; thus, it can serve as a scaffold material to prepare a nerve conduit to provide effective support for cells and induce the proliferation, differentiation and migration of nerve cells, as well as a controlled drug release system to further improve the rate of nerve regeneration. Therefore, LBP/NGF-loaded anisotropic conductive nanofiber scaffolds can be applied to tissue engineering for the treatment of nerve injury.
